# Assessing the STEM landscape: the current instructional climate survey and the evidence-based instructional practices adoption scale

**DOI:** 10.1186/s40594-017-0092-1

**Published:** 2017-11-15

**Authors:** R. Eric Landrum, Karen Viskupic, Susan E. Shadle, Doug Bullock

**Affiliations:** Boise State University, Boise, ID USA

**Keywords:** Instructional climate, Measurement, STEM, Evidence-based instructional practices

## Abstract

**Background:**

The efficacy of active learning within STEM education is clear, and many institutions are working to help faculty adopt evidence-based instructional practices (EBIPs) which can promote active learning. In order to know the current status of our campus regarding these goals, measures of current instructional climate and the adoption of evidence-based instructional practices (EBIPs) are desired.

**Results:**

Using a campus-wide online survey approach with remuneration for faculty participants, the 28-item current instructional climate survey (CICS) and the 6-item EBIP adoption scale were developed. When CICS and EBIP adoption scale outcomes are compared, patterns emerge which reflect the climate, conditions, and personal characteristics of faculty at different stages of EBIP adoption.

**Conclusions:**

Although not causal relationships, understanding both climate and personal change characteristics can be helpful to campus change agents in assessing the current STEM landscape of faculty practices.

**Electronic supplementary material:**

The online version of this article (10.1186/s40594-017-0092-1) contains supplementary material, which is available to authorized users.

When staff and faculty operate from routines, change can be challenging. Imagine trying to have STEM faculty move from lectures to active learning. Their underlying belief is that good teaching involves delivery of content. Asking them to move to a mode where they do not deliver content violates their unarticulated beliefs about good teaching. Cultural theories of change emphasize the need to analyze and be cognizant of these underlying systems of meaning, assumptions, and values; while often not directly articulated, they can nonetheless shape institutional operations and prevent or facilitate change (Kezar and Holcombe 2016, p. 38).

For widespread adoption of evidence-based instructional practices (EBIPs) to occur, the complex higher education ecosystem must be altered; it is important for institutional operations and instructional climate to be understood (Association of American Universities 2017; Rankin and Reason 2008). For many faculties in the USA, lecturing remains widespread, with 50.6% of professors indicating a reliance on extensive lecturing (Eagan et al., 2014). This reliance is understandable, as many faculty members teach as they were taught, but this level of reliance is also surprising given the emerging empirical data about the benefits of active learning. Freeman et al., (2014), using a meta-analysis of 225 STEM education research studies, concluded that active learning approaches are robustly superior in regard to reducing course failure rates and increasing student learning in STEM disciplines; Wieman (2014) has referred to lecturing as “…the pedagogical equivalent of bloodletting” (p. 8320). Given the increasing pressures to transform institutions in regard to undergraduate STEM education (Weaver et al., 2016) and the understanding that changing teaching behaviors is personal and difficult to achieve (Andrews and Lemons 2015), those institutions attempting institutional change would benefit from measures of instructional climate (Adams Becker et al., 2017) as well as indicators of the adoption of active learning approaches. To fully understand how STEM faculty make changes to their teaching practices, the instructional climate is one of the key indicators; in fact, Kober (2015) concluded that “…a lack of attention to the larger institutional context is one reason why research-based practices in undergraduate science and engineering education have not produced more widespread change, despite evidence of their effectiveness” (p. 177). The ability to assess the current teaching landscape could be an important ally in these efforts. That is, if the subjective norm of the environment becomes teaching via EBIPs rather than lecture, according to Ajzen’s (1991) theory of planned behavior faculty members who remain in lecture mode will be more inclined to change given the new environmental conditions. If there is a tipping point (Gladwell 2002) for faculty in STEM departments to be predisposed to transforming their teaching practices toward more active learning approaches, it would be helpful to have measures of instructional climate available so that campus leaders can leverage prevailing trends and ensure adequate support for faculty members in every EBIP adoption stage.

## Measuring instructional climate

Measuring the instructional climate of a college or university is important if the desired goal is to create and measure transformational change around teaching and learning. In fact, the values of an organization, including its underlying assumptions, are key drivers of and barriers to change (Kezar and Holcombe 2016). There is no shortage of available instruments for measuring teaching practices, either by using self-report surveys (e.g., Postsecondary Instructional Practices Survey (Walter et al., 2016) and Teaching Practices Inventory (Wieman and Gilbert 2014), or observation protocols (e.g., Reformed Teaching Observation Protocol (Piburn et al., 2000) and Classroom Observation Protocol for Undergraduate STEM (Smith et al., 2013)). However, our interest is in the faculty perceptions of the instructional climate, which includes more than the pedagogies selected for use. Even though there are measures of instructional climate that exist in the literature in various forms (e.g., measuring departmental climate from Walter et al., (2016)), our desire was to create a climate measure that was (a) specific to the instructional climate of a university and (b) designed to measure the climate elicited from a specific organizational change process/theory. Literally, climate is a local phenomenon, and thus, it seemed logical to develop a local instrument, but also to be vigorous in establishing the validity and reliability of its measures.

Many change models exist, such as the Gess-Newsome et al., (2003) model for faculty change and the Henderson et al., (2011) four-quadrant model of strategies for change. We utilized Dormant’s (2011) CACAO (Change, Adopters, Change Agent, and Organization) model because of our familiarity with the model and access to local experts in using this model (see also Shadle et al., 2017). The CACAO model is rooted in Rogers (2003) diffusion of innovation theory that specifically outlines a set of actions that can be taken to facilitate change. In the context of the present study, implementation of the CACAO change model is the intervention strategy that has been used to facilitate the adoption of active learning practices by faculty members.

## Evidence-based instructional practice adoption stages

With the presumption that there are steps or stages of change through which faculty move as they adopt new teaching approaches, it would be useful to know a faculty member’s particular status within the continuum of change; a one-size-fits-all intervention strategy is unlikely to be universally successful when STEM faculty members vary in their readiness to adopt. To this end, we used a Guttman scaling approach to develop our EBIP adoption scale. Well-developed Guttman scales are inherently intuitive because (1) responses are merely yes or no and (2) scoring is easy and obvious by examining when/where the pattern of responses changes. Although there are multiple good survey inventories available in the literature where faculty members describe their usage of pedagogical practices (PIPS, TPI), to our knowledge, there is no existing measure that allows a faculty member to self-identify their level or stage of adoption of evidence-based instructional practices. That is, a teaching practices inventory may help a faculty member report that their predominant teaching pedagogy is lecture, but that same inventory does not yield information about that faculty member’s thoughts about alternative EBIP strategies, whether they have imagined using an EBIP in their course, whether they have attended a workshop about adopting a new EBIP, and so on.

In the change model, Dormant (2011) suggested five levels or stages of the potential adopter, described here: (1) Awareness: The potential adopter is passive about the change, has little/no information about the change, and has little/no opinion about the change; (2) Curiosity: The potential adopter wants more information about the change, actively engages in asking questions about the change, and asks questions about personal impact; (3) Mental tryout: The potential adopter is in a pre-commitment stage, imagining how the change would be made, asking job-focused questions (with job-focused concerns) about the impact of the change; (4) Hands-on tryout: The potential adopter has made the commitment to change, wants to learn how to implement the change, has opinions about the change, and asks questions about the change relative to organizational context; and (5) Adoption: The potential adopter has now actively made the change, is able to make suggestions for improvement regarding the change, and may seek out expert opinion for answers to detailed questions about the change. Although not specifically articulated in Dormant’s model, if awareness is stage 1, there could essentially be a stage 0, that is, a pre-awareness stage.

Given the goal of changing STEM faculty adoption of active learning, it would be valuable to know the current stage of a faculty member, and perhaps also the cumulative status of a department. A faculty member who is unaware of EBIPs will need a different level of support and training than a faculty member who is a long-time adopter of EBIPs; for faculty developers and campus change agents, one size (intervention) does not fit all. Departmental context is also an important factor to consider when attempting to change faculty teaching behaviors (Lund and Stains 2015; Manduca et al., 2017). The intervention strategies implemented by campus change agents for those in the awareness and curiosity stages should certainly be different compared to those intervention strategies implemented for faculty members in the hands-on tryout or adoption stages (Dormant 2011). An understanding of the adoption stage, paired with current instructional climate data, could provide change agents with useful information about which faculty and departments are most ready for intervention efforts. Given this context, our research questions include: (1) When attempting to measure the construct of instructional climate, what are the reliable and valid components or factors that emerge? (2) Can a straightforward scale be developed that allows STEM faculty to meaningfully self-identify their own adoption stage regarding the usage of evidence-based instructional practices? and (3) How are measures of instructional climate and EBIP adoption stage useful to campus leaders, and how might these measures be related to existing demographic variables that describe the sample?

## Method

### Participants

In order to understand institutional climate and adoption stages, all Boise State University faculty with teaching responsibilities (*N* = 1799) during the Fall 2015 and Spring 2016 semesters were surveyed in 2016; respondents received $10.00 remuneration placed directly on their campus identification card. To qualify as a faculty member with teaching responsibilities, the following criteria were utilized: (a) the faculty member had to be listed as teaching at least one course in the Registrar’s database and (b) the course must have an enrollment greater than one (which allowed ruling out independent study/thesis type courses). This method generated a comprehensive list of instructors, including graduate students, adjunct faculty, tenure and non-tenured full-time faculty, administrators with a teaching appointment, off-campus instructors, and online instructors. With 528 usable responses, the overall response rate was 30.1%.

### Materials

#### Development of the current instructional climate survey

We used Dormant’s (2011) change process/protocol in order to engage faculty members in thinking about an end state on our campus (Shadle et al., 2017) that would look like this:

The culture of teaching and learning at Boise State will be characterized by an on-going exploration and adoption of evidence-based instructional practices which includes (a) faculty engaged in continuous improvement of teaching and learning; (b) dialog around teaching supported through a community of practice; and (c) teaching evidenced and informed by meaningful assessment. The fulfillment of this vision will result in increased student achievement of learning outcomes, retention, and degree attainment, especially among underrepresented populations.

Working with two groups of STEM faculty based on convenience sampling, we engaged these faculty members to describe the positive and negative aspects of moving toward the desired end state. Faculty responded on paper surveys in each of the five key characteristic areas in regard to achieving the goal state (relative advantage, simplicity, compatibility, adaptability, and social impact; Dormant 2011). Based on pilot testing from the CACAO-based change adoption process and with the aid of a survey design expert, we organized responses using a modified Q-sorting technique (see Nitzberg (1980) and DeNelsky and McKee (1969) for more Q-sort examples) to identify thematic trends. From these empirically-derived themes, we generated the initial item pool for the current instructional climate survey (CICS). For example, faculty noted “a sense of central administration taking over” as a potential barrier to changing instructional practices. In response, we crafted a semantic differential item with the stem “I believe that the campus culture…” with the anchors ranging from “limits the choice of teaching methods” to “allows for the free choice of teaching methods.” Thus, each item in the CICS was based on this analysis of the positive and negative aspects of the potential change (i.e., drivers and barriers) in working toward the desired end state, see Tables 1, 2, and 3 for the CICS items. Items were pilot-tested and re-tested until the resulting pool of 28 items was finalized. It is important to emphasize that all of the items generated for this work originated from STEM faculty members.

**Table 1:** Campus climate

Item no.	Item	M (SD)	Item
1	is generally supportive of teaching.	2.62 (1.5)	is generally unsupportive of teaching.
2	limits the choice of teaching methods.	5.48 (1.5)	allows for the free choice of teaching methods.
3	promotes faculty-centered teaching.	4.56 (1.5)	promotes student-centered teaching.
4	values research more than teaching.	3.38 (1.7)	values teaching more than research.
5	is student-success oriented.	2.98 (1.5)	is not student-success oriented.
6	connects me with other teachers.	3.50 (1.6)	isolates me from other teachers.
7	does not value teaching ability in hiring decisions.	4.29 (1.7)	does value teaching ability in hiring decisions.
8	discourages me from trying new teaching techniques.	5.48 (1.5)	encourages me to try new teaching techniques.
9	values the assessment of student learning outcomes.	2.92 (1.6)	does not value the assessment of student learning outcomes.
10	values teaching more than research in tenure and promotion decisions.	5.18 (1.5)	values research more than teaching in tenure and promotion decisions.
11	is shaped by leaders who are not supportive of my teaching.	4.83 (1.5)	is shaped by leaders who are supportive of my teaching
12	encourages use of evidence-based instructional practices	2.77 (1.4)	discourages use of evidence-based instructional practices
13	does not value teaching.	5.22 (1.5)	values teaching.
14	does not allow faculty to teach using any method they choose.	5.55 (1.3)	allows faculty to teach using any method they choose.
15	breeds divisiveness in teaching discussions.	5.12 (1.4)	breeds collaborative teaching discussions.
16	is characterized by high faculty-student rapport.	3.12 (1.4)	is characterized by low faculty-student rapport.

For this seven-point semantic differential scale, the left-most response was coded 1 and the right-most response was coded 7. Individual item Ns vary from 516 to 536

Means (M) and standard deviations (SD) for current instructional climate survey (CICS) items

For each item, please select the scale point that best represents your opinion. Each statement begins with “I believe that the campus culture…”

**Table 2:** My teaching

Item no.	Item	M (SD)	Item
17	faculty-centered.	5.84 (1.2)	student-centered.
18	unmonitored.	3.78 (1.8)	monitored.
19	a small part of my professional identity.	5.52 (1.5)	a large part of my professional identity.
20	not valued.	5.25 (1.5)	valued.
21	more important than my research.	3.58 (1.8)	less important than my research.
22	not informed by discussions with colleagues.	5.30 (1.5)	informed by discussions with colleagues.
23	less important than my research when I am considered for tenure and promotion.	3.10 (1.5)	more important than my research when I am considered for tenure and promotion.
24	not informed by research about best practices.	5.58 (1.2)	informed by research about best practices.

For this seven-point semantic differential scale, the left-most response was coded 1 and the right-most response was coded 7. Individual item Ns vary from 499 to 532

Means (M) and standard deviations (SD) for current instructional climate survey (CICS) items

For each item, please select the scale point that best represents your opinion. Each statement begins with “I believe that my teaching is…”

**Table 3:** My institution

Item no.	Item	M (SD)
25	adequate resources to support teaching.	3.82 (1.0)
26	flexible, physical spaces for teaching and learning.	3.38 (1.1)
27	adequate mechanisms for evaluating teaching.	3.10 (1.1)
28	adequate assessment mechanisms/support.	3.32 (1.0)

Individual item Ns vary from 529 to 532

Means (M) and standard deviations (SD) for current instructional climate survey (CICS) items

For each item, please select the scale point that best represents your level of agreement, with 1 = strongly disagree, 2 = disagree, 3 = neutral, 4 = agree, and 5 = strongly agree. Each statement begins “I believe that my institution provides…”

The first 24 items of the CICS were answered on a 1 to 7 semantic differential scale, as described above. Another example of this type of scaled item “I believe that the campus culture…” with the low (value = 1) anchor being “connects me with other teachers” and the high (value = 7) anchor being “isolates me from other teachers.” The remaining four items of the CICS were answered using a Likert-type agreement scale from 1 = strongly disagree to 5 = strongly agree. After pilot testing, the nature of these items appeared to be better answered on an agreement scale rather than a semantic differential scale. Examples from this last section of the CICS include the stem of “I believe that my institution provides…” and items such as “flexible, physical spaces for teaching and learning” and “adequate assessment mechanisms/support.”

#### Development of the evidence-based instructional practices adoption scale

The items in the EBIP adoption scale were developed, a priori, to be used as a Guttman scale with yes/no responses. Our goal was to generate at least one yes/no question for each of the five CACAO adoption stages (Dormant 2011). Members of the research team, working with a survey expert, generated a pool of Guttman scale (yes/no) items that comprised the initial item pool for pilot testing. After pilot testing, one item was selected to map onto each stage of the CACAO change model. One of the objectives of a Guttman scale is unidimensionality, that is, the measure of a singular construct—in the present case, this singular dimension is the faculty members’ degree of adoptions of EBIPs.

Self-scoring of Guttman scales is evident when the pattern of responses changes from yes to no. This goal is operationalized in the calculation of the coefficient of reproducibility (CR); a CR = 1.0 would indicate a perfectly replicable Guttman scale. In practice, a CR > .90 is considered the standard of evidence for unidimensionality (Abdi 2010; Aiken and Groth-Marnat 2006; Guest 2000). However, if extreme patterns of responses to an item emerge or an individual responds with an extreme pattern (e.g., answering all of the items with *yes*), these types of patterns can lead to an artificially high CR (Guest 2000; Menzel 1953). To counteract this, Menzel (1953) developed the coefficient of scalability (CS), “…which measures predictability of the scale relative to the level of prediction afforded by consideration solely of the row and column totals” (p. 351). The recommended standard for a CS is .60 (Guest 2000; Menzel 1953).

Following the formulation and pilot testing of the Guttman scale items, this new instrument was administered to 528 participants at the same time of the CICS item administration, see Additional file 1: Table S1 for the seven EBIP adoption scale items. Following data collection, responses were assembled and ordered from most agreement (highest number of *yes* responses) to least agreement (lowest number of *yes* responses). For each item, scale errors were calculated following Aiken and Groth-Marnat (2006) and Guest (2000) and marginal errors (i.e., non-modal frequencies) were calculated according to the methods suggested by Guest (2000) and Menzel (1953). Any participant who left a Guttman item blank was eliminated from the analysis (*N* = 14); this resulted in the data from 514 respondents utilized for the Guttman scale analysis.

Similar to the process of eliminating items from a scale to increase inter-item reliability as evidenced by a Cronbach’s *α*, Guttman scale items were systematically tested in order to achieve adequate levels of reproducibility and scalability. Ultimately, the original item #2 was removed from the initial seven items, and this process resulted in a six-item scale (see Additional file 1: Table S1) with a CR = .931 and a CS = .792. This process is similar to using inter-item coefficients when testing the Cronbach’s *α* of Likert-type subscales; removal of the original item #2 allowed for the resulting Guttman item pool to reach acceptable reliability.

#### Demographics

The demographic questions included faculty rank, total years teaching experience in higher education and at Boise State, the year graduated with their highest academic degree, the highest academic degree in one’s primary discipline, the primary academic department or unit, tenure/tenure track or non-tenure track, age, gender, whether or not the faculty member has an office on campus, an approximation of one’s normal workload that involves teaching and research, and institutional identification number (this was necessary in order to remunerate participants for survey completion), see Additional file 2: Table S2 for the demographic characteristics of the overall sample.

### Procedure

At the end of January 2016, all Boise State faculty with teaching responsibilities were invited via E-mail to complete the current instructional climate survey (CICS), the Postsecondary Instructional Practices Scale (PIPS; Walter et al., 2016), the EBIP Adoption Scale, and demographic questions. The PIPS items are not analyzed as part of the current study. All measures were administered online via Qualtrics. Survey participation closed at the end of February and during the time the survey was available; two follow-up reminders were E-mailed to non-respondents only. Respondents could take as much time as they wanted to reply to survey items. Respondents received $10 placed directly on their university identification card.

## Results and discussion

This section is subdivided based on the outcomes of the development of the CICS and the EBIP Adoption Scale, including subsections on descriptive outcomes, CICS factor analysis results, climate and adoption scale results considered together, factor analysis results, and analyses based on select demographic variables. A discussion of each of the outcomes is included here for clarity, followed by a Conclusions section.

### Descriptive outcomes for the CICS and EBIP adoption scale

For the overall means and standard deviations for all of the CICS survey items, see Tables 1, 2, and 3. Note that for the first two sections of the CICS, each item was answered on a seven-point semantic differential scale, with the left-most response coded as 1 and the right-most response coded as 7. For example, for the item “I believe that the campus culture (‘does not value teaching’ to ‘values teaching’),” a lower score means that faculty responses were closer to the left-most “does not value teaching” anchor (1), and a higher score means that faculty responses were closer to the right-most “values teaching” anchor (7), with an exact midpoint at 4.0. For this particular item, the mean response value was 5.22 (SD = 1.5), meaning that across all faculty respondents, on average, they tend to believe that the campus culture values teaching. With regular and meaningful measurement, answers to particular items can be helpful. For instance, observing relatively high values on the initial measurement can inform researchers that the current campus climate on a particular issue is highly positive; given this observation, efforts to significantly increase perceptions may be difficult due to ceiling effects.

The descriptive outcomes for the EBIP adoption scale responses consist of scale scores and how they map onto Dormant’s (2011) CACAO change model adoption stages. For these results, see Additional file 1: Table S1. This type of measure could be particularly valuable over time, as shifts in departmental culture can be tracked based on the distribution of faculty across different stages of EBIP adoption.

### Factor analytic outcomes for the CICS

All responses to the 28-item CICS, items were subjected to exploratory factor analysis using a varimax rotation, eigenvalues > 1.25, and factor loadings > 50. A five-factor solution emerges explaining 54.1% of the variance.

The theme that emerges for factor 1 (items 14, 2, 8, and 15; see Tables 1, 2, and 3) is the free choice of teaching methods, which involves the encouragement of using new teaching methods as well as collaborative discussions; inter-item reliability using Cronbach’s *α* = .797. The higher the factor 1 score, the greater the belief that the free choice of teaching methods exists. The theme for factor 2 (items 27, 28, 26, and 25) is institutional support, meaning that there is adequate support for teaching, assessment, evaluation, and the availability of physical, flexible spaces for teaching; inter-item reliability using Cronbach’s *α* = .805. The higher the factor 2 score, the greater agreement that there is institutional support for teaching. The theme for factor 3 (items 10, 4, and 23, with reverse coding for item 10) is teaching-research balance, including the relative valuing of teaching and research in hiring as well as promotion and tenure decisions; inter-item reliability using Cronbach’s *α* = .759. The higher the score for factor 3, the more that teaching is valued over research, including hiring and promotion and tenure decisions. The theme for factor 4 (items 9 and 12, with both items reverse-coded) is the encouragement to use evidence-based instructional practices, especially as related to assessing student learning outcomes; inter-item reliability using Cronbach’s *α* = .619. The higher the score for factor 4, the greater the belief that the campus climate encourages the use of evidence-based instructional practices. Lastly, the theme for factor 5 (items 22 and 6, with item 6 reverse-coded) is teacher connectedness, involving the connections and conversations with teaching colleagues; inter-item reliability using Cronbach’s *α* = .615. The higher the score for factor 5, the greater connectedness with teaching colleagues, especially as related to teaching discussions. Even though the inter-item reliabilities are low for factor 4 and factor 5, they were retained here for explanatory purposes.

### Combination of climate and adoption stage: CICS factor scores and EBIP adoption scale outcomes

Scores from the five CICS factor scores were correlated with EBIP adoption scale scores. Due to five correlation coefficients being generated, a Bonferroni correction was employed to minimize family-wise error. The resulting *p* critical value (*p*_crit_) is .01. EBIP adoption scale scores are significantly correlated with (a) factor 1 (the free choice of teaching methods), *r*(531) = .13, *p* = .004; (b) factor 3 (teaching-research balance), *r*(531) = −.18, *p* < .001, (c) factor 4 (encouragement to use evidence-based instructional practices), *r*(528) = .14, *p* = .002, and (d) factor 5 (teacher connectedness), *r*(531) = .22, *p* < .001. What does this mean? The higher the self-reported stage on the EBIP adoption scale (a) the greater the perception of free choice in teaching, (b) the greater the weighting of teaching in considering teaching-research balance, (c) the greater the perceived encouragement on campus to use evidence-based instructional practices, and (d) the more connected the faculty member feels to other teachers on campus.

### Select demographic variables as related to CICS scores

For all of the CICS-related analyses in this section, the Bonferroni correction was used for the five comparisons, resulting in *p*_crit_ = .01.

#### Age

Answers to the items which comprise factor 1 (the free choice of teaching methods) were significantly correlated with age, *r*(493) = .15, *p* = .001. Younger faculty reports greater freedom to select the teaching method of their choice. Answers to the items which comprise factor 3 (teaching-research balance) were significantly correlated with age, *r*(493) = −.12, *p* = .008. With the negative correlation, younger faculty members report their belief that research is valued over teaching in the teaching-research balance.

#### Teaching workload

Respondents were asked to report the approximate percentage of their workload that involves teaching. There is a significant correlation between responses to factor 3 (teaching-research balance) and responses to the teaching workload item, *r*(526) = .13, *p* = .002. Faculty members reporting higher workload percentages for teaching perceive teaching is more valued in hiring decisions and promotion and tenure decisions.

#### Tenure/tenure track vs. non-tenure track

When the responses are compared between tenure/tenure-track faculty and non-tenure-track faculty, significant differences emerge for two CICS factors: (1) tenure/track faculty (mean = 3.18, SD = 0.9) score significantly lower than non-tenure-track faculty (mean = 3.53, SD = 0.8) on factor 2 (institutional support), *t*(526) = −4.76, *p* < .001 and (2) tenure/tenure-track faculty (mean = 2.58, SD = 1.3) score significantly lower than non-tenure-track faculty (mean = 3.45, SD = 1.2) on factor 3 (teaching-research balance), *t*(526) = −7.89, *p* < .001. Tenured/tenure-track faculty believe there is less institutional support for teaching compared to non-tenure-track faculty, and tenured/tenure-track faculty believe that research is more valued over teaching as compared to the balance perceived by non-tenure-track faculty.

#### Office on campus

For the CICS factor scores, there were three significant differences in answers between those individuals with an office on campus and not having an office on campus: (1) individuals with an office (mean = 3.33, SD = 0.8) scored significantly lower than individuals with no office (mean = 3.68, SD = 0.8) on factor 2 (institutional) support, *t*(530) = −4.07, *p* < .001; (2) individuals with an office (mean = 2.96, SD = 1.3) scored significantly lower than individuals with no office (mean = 3.73, SD = 1.1) on factor 3 (teaching-research balance), *t*(530) = −5.91, *p* < .001; and (3) individuals with an office (mean = 5.00, SD = 1.3) score significantly higher than individuals with no office (mean = 4.52, SD = 1.4) on factor 5 (teacher connectedness), *t*(530) = 3.64, *p* < .001. When answers to the office on campus item are compared to academic status (non-tenure track vs. tenure/tenure track), there is a significant association in the pattern of answering these two items, *X* ^2^(1) = 75.99, *p* < .001; 98.5% of tenure/tenure-track faculty members have an office on campus compared to 66.1% of non-tenure-track faculty members.

Faculty members with an office on campus actually believe that there are fewer institutional resources for teaching compared to those faculty without offices on campus. Faculty members without an office believe that the institution values teaching over research more than faculty members with an office. Lastly, faculty members with an office report greater connectedness to other teachers on campus compared to those faculty without offices on campus.

#### Gender

There were no significant differences between male and female responses on each of the five CICS factors.

### Demographic variable relationships with EBIP adoption scale scores

EBIP adoption scores were significantly correlated with answers to the item about the percentage of workload involving research, *r*(367) = −.12, *p* = .027; with the negative correlation coefficient, the less workload involving research, the higher the EBIP adoption score. There is a significant difference between tenure/tenure-track faculty (mean = 3.82, SD = 2.0) and non-tenure-track faculty (mean = 3.42, SD = 2.2) on their EBIP adoption scores, *t*(526) = 2.08, *p* = .038; tenure-track faculty report significantly higher EBIP adoption scores. There is a significant difference in answers for those individuals with an office (mean = 3.75, SD = 2.1) and those individuals who do not have an office (mean = 2.91, SD = 2.3) on EBIP adoption scores, *t*(530) = 3.84, *p* < .001; those with an office report higher EBIP adoption score. Also, there is a significant difference between females (mean = 3.86, SD = 2.1) and males (mean = 3.18, SD = 2.1) on EBIP adoption scores, *t*(498) = −3.51, *p* < .001; females report higher EBIP adoption scores than males.

#### EBIP departmental profiles

**Fig. 1:**
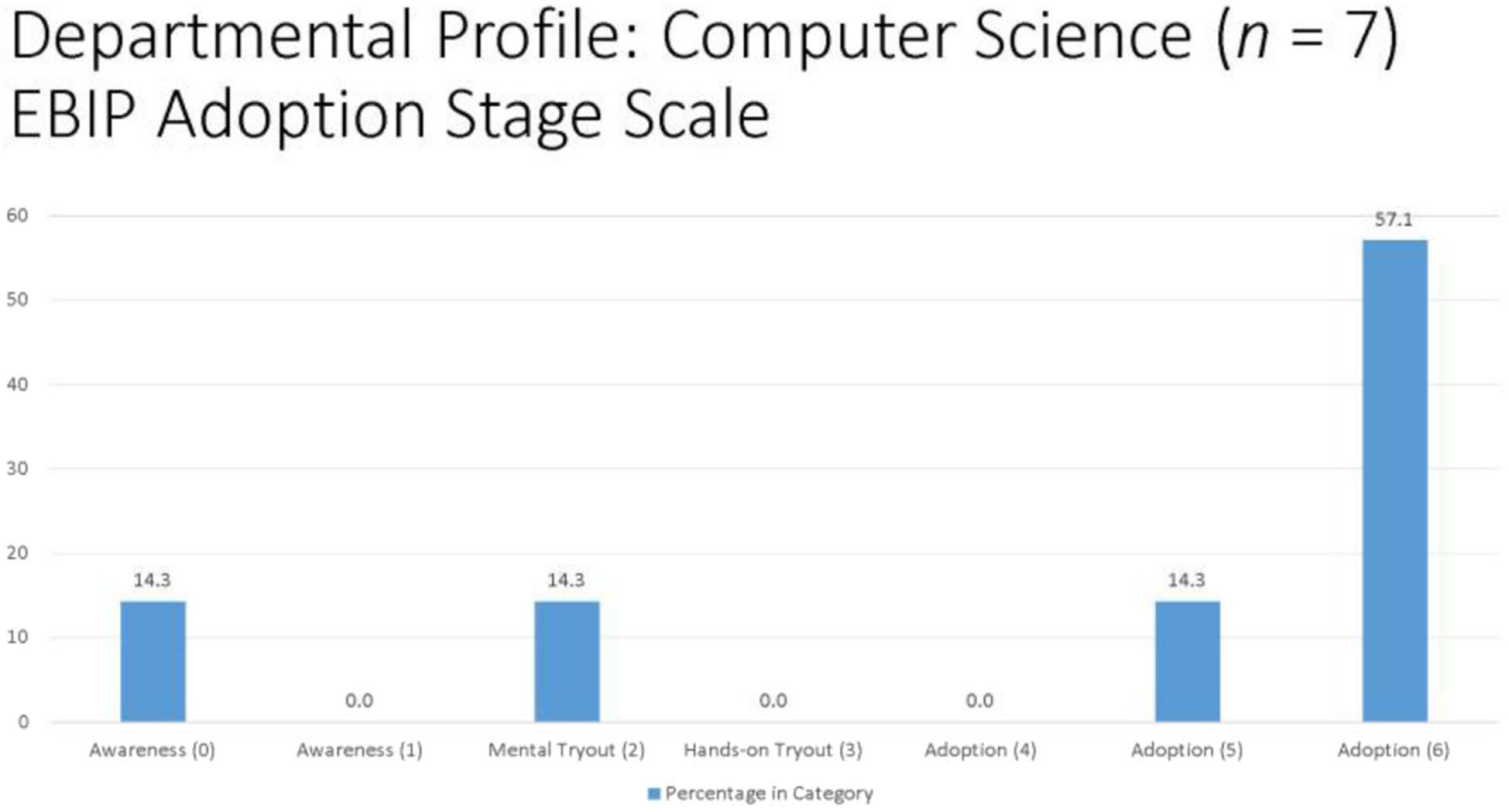
Departmental Profiles Based on EBIP Adoption Stage Scale Scores

**Fig. 2:**
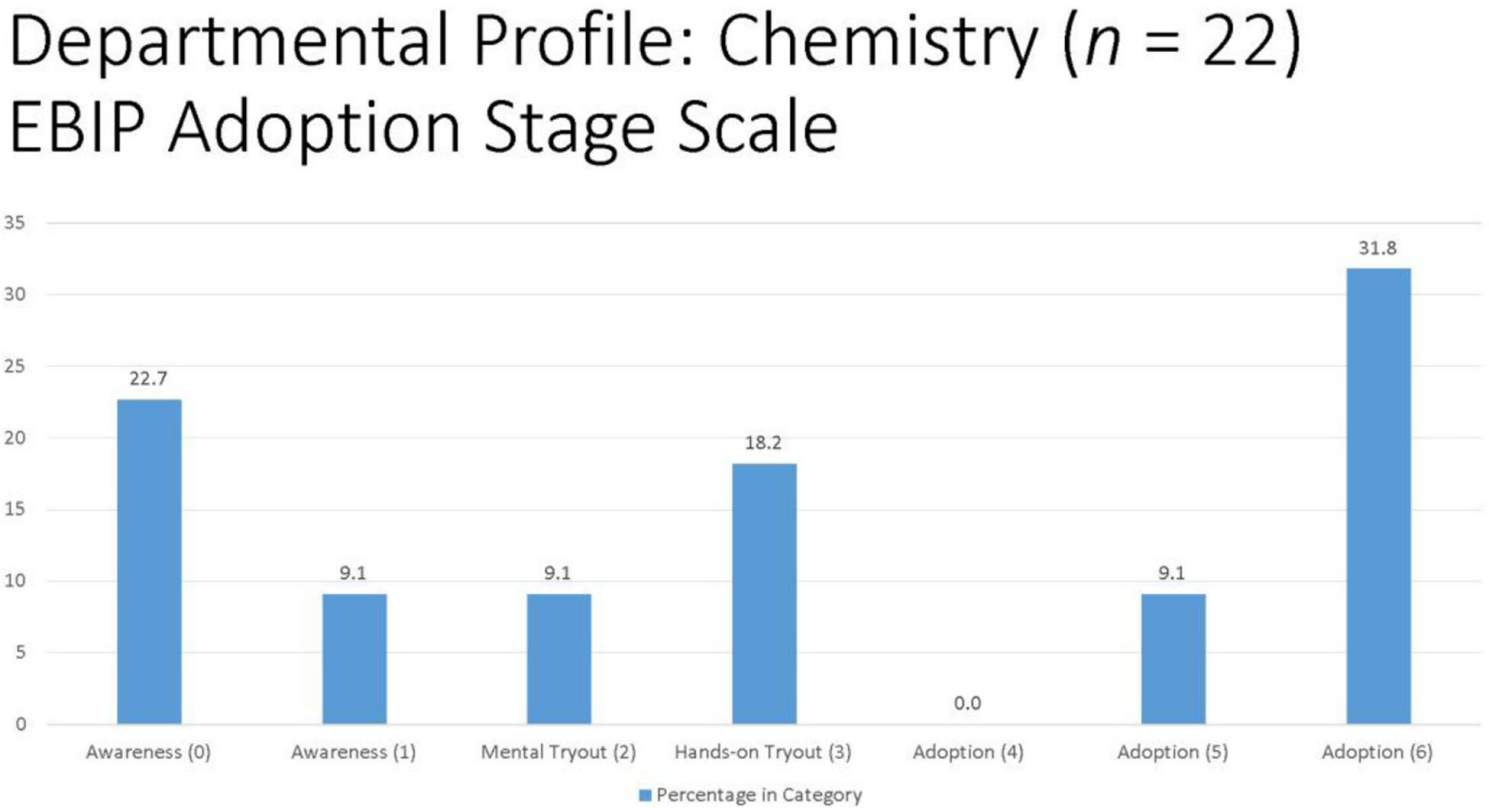
Departmental Profiles Based on EBIP Adoption Stage Scale Scores

With the existence of individually based EBIP adoption scores, departmental profiles can be created to depict the climate or culture within a department concerning the adoption of evidence-based instructional practices. There are strong advocates for changes in STEM education (Freeman et al., 2014; Wieman 2014), and utilizing EBIP departmental profiles for STEM departments could provide a new measure of assessing the landscape. Following the calculation of EBIP scores in the current study, departmental profiles were created for each of the STEM departments under study, see Fig. 1 for examples of STEM department profiles. By reviewing the departmental profiles such as in chemistry or computer science, campus leaders interested in the transformation of both faculty practice and institutional climate may realize that a one-size-fits-all approach in encouraging faculty members to adopt evidence-based instructional practices will likely not work. For instance, multiple strategies for EBIP adoption are needed in Chemistry due to the diversity of scores on the EBIP adoption scale (Fig. 2). However, campus leaders might decide to provide more resource-intensive support to Computer Science since the bulk of respondents are already EBIP adopters.

It is clear to see that different faculty members are aligned at different points on the EBIP Adoption Scale; thus, strategies for those individuals at the awareness stage should be different than the strategies needed for those in the mental tryout or adoption stages. Department profiles could be a powerful source of information for campus leaders in determining tipping points for localized, grassroots efforts to affect teaching practices.

## Conclusions

As for limitations, this is a single sample from one institution of higher education; greater use among more and diverse educational institutions would help to re-affirm the reliability of the initial findings presented here. To that end, the specific items that comprise the CICS are shared in Tables 1, 2, and 3, and those of the EBIP adoption scale are in Additional file 1: Table S1, with the goal of facilitating expanded work by other researchers where interested; our team will continue to use this instrument and continue to explore the case for its beneficial use. There are also subtle distinctions between measuring the instructional climate of an institution as compared to faculty members’ perceptions of the climate. In the present case, perception may be reality; that is, Kober (2015) and Kezar and Holcombe (2016) would argue that understanding the values and the institutional context are vital to the understanding of change and transformation.

The CICS has become a valuable tool in our applied work with STEM departments because it allows for an assessment of the current institutional climate regarding teaching and how it is perceived, valued, and supported on campus. The five-factor structure of this scale makes sense and its use in statistical analyses has already allowed for meaningful insights for our applied work. The overarching goal is for an EBIP adoption scale score to serve as an index of an individual STEM faculty member’s placement on the adoption scale as described previously by Dormant (2011) and as adapted here, specifically, for the use of evidence-based instructional practices.

This is a challenging era in higher education; a growing focus on assessment, accountability, student learning and student success is underway. Change will happen, voluntary or otherwise (i.e., innovation or stagnation). Institutions will either effect strategic, planned transformation in alignment with national and regional goals or have it forced upon them. To this end, it would be advantageous to have meaningful measures in place in order to assess the current STEM landscape regarding instructional climate and the adoption of evidence-based instructional practices. The development of such measures is the precise focus of this study, more specifically, to develop a measure of current instructional climate and EBIP adoption stage. Based on our initial findings, the CICS appears to be a useful measure to provide campus leaders with a current “snapshot” of STEM faculty attitudes, beliefs, and behaviors regarding teaching. The EBIP adoption scale allows for the identification of an adoption stage for STEM faculty members, and that information can be useful in designing effective interventions to meet faculty members where they are, and for monitoring changes in faculty EBIP adoption and use over time. We encourage researchers to use these instruments in order to foster a greater understanding of instructional climate as well as EBIP adoption stages for individuals and group from diverse institutional contexts.

## Additional files

Additional file 1: Table S1. EBIP adoption scale item development (Groccia and Buskist (2011). (DOCX 15 kb)

Additional file 2: Table S2. Demographic outcomes. (DOCX 14 kb)
