# Supplementary material for: Assessing the STEM landscape: the current instructional climate survey and the evidence-based instructional practices adoption scale
Source: Int J STEM Educ. 2017 Nov 15;4(1):25. doi: 10.1186/s40594-017-0092-1 (PMC6310375; doi:10.1186/s40594-017-0092-1)
Supplement: Supplementary file 1 — EBIP adoption scale item development (Groccia and Buskist (2011). (DOCX 15 kb) [file 40594_2017_92_MOESM1_ESM.docx]

Additional file 1: Table S1

*EBIP Adoption Scale Item Development*

In the box below is how the items were presented to participants:

| **Instructions**. Please read the following definition of EBIP and answer Items 1-6 with a yes or no response.  What is an EBIP?  It is an *evidence-based instructional practice* or approach that has a demonstrated record of success.  That is, there is reliable, valid empirical evidence to suggest that when instructors use EBIPs, student learning is supported, and it is implied that EBIPs are more effective than standard traditional lecture and discussion methods (Groccia & Buskist, 2011).  Active learning techniques are often EBIPs, such as just-in-time teaching, process oriented guided inquiry learning, think-pair-share, cooperative learning, peer instruction, service learning, and many others.   \| Scale Items \| Yes \| No \| \| --- \| --- \| --- \| \| 1. Prior to this survey, I already knew about evidence-based instructional practices (EBIPs). \| O \| O \| \| 1. I am curious about how my teaching would change if I used more EBIPs. \| O \| O \| \| 1. I have thought about how to implement EBIPs in my courses. \| O \| O \| \| 1. I’ve spent time learning about EBIPs (e.g., attended workshop, experimented in class, read education literature) and I am prepared to use them. \| O \| O \| \| 1. I consistently use EBIPs in my courses. \| O \| O \| \| 1. I consistently use EBIPs and I continue to learn about and experiment with new EBIPs. \| O \| O \| \| 1. I have evidence that my teaching has improved since I started using EBIPs. \| O \| O \| |
| --- | --- | --- | --- | --- | --- | --- | --- | --- | --- | --- | --- | --- | --- | --- | --- | --- | --- | --- | --- | --- | --- | --- | --- | --- |

Final EBIP Adoption Scale Items

1. Prior to this survey, I already knew about evidence-based instructional practices (EBIPs).
2. I have thought about how to implement EBIPs in my courses.
3. I’ve spent time learning about EBIPs (e.g., attended workshop, experimented in class, read education literature) and I am prepared to use them.
4. I consistently use EBIPs in my courses.
5. I consistently use EBIPs and I continue to learn about and experiment with new EBIPs.
6. I have evidence that my teaching has improved since I started using EBIPs.

Scoring

| Score  (number of YES responses) | CACAO Change Model  (Dormant, 2011) | Number of Individuals  with This Score |
| --- | --- | --- |
| 0 | Awareness | 78 |
| 1 | Awareness | 38 |
| 2 | Mental Tryout | 47 |
| 3 | Hands-on Tryout | 60 |
| 4 | Adoption | 39 |
| 5 | Adoption | 128 |
| 6 | Adoption | 124 |
