# Supplementary material for: Assessing the STEM landscape: the current instructional climate survey and the evidence-based instructional practices adoption scale
Source: Int J STEM Educ. 2017 Nov 15;4(1):25. doi: 10.1186/s40594-017-0092-1 (PMC6310375; doi:10.1186/s40594-017-0092-1)
Supplement: Supplementary file 2 — Demographic outcomes. (DOCX 14 kb) [file 40594_2017_92_MOESM2_ESM.docx]

Additional file 2: Table S2

*Demographic Outcomes*

Faculty Rank Responses (Note: Participants could select more than one category)

|  | Number Responding |
| --- | --- |
| Adjunct faculty | 147 |
| Administrator | 5 |
| Assistant professor | 65 |
| Associate professor | 63 |
| Clinical faculty | 13 |
| Emeritus faculty | 1 |
| Full professor | 51 |
| Graduate student | 56 |
| Lecturer | 72 |
| Visiting professor | 6 |

Teaching Experience, Age, and Workload

|  | Means (*SD*) |
| --- | --- |
| Total Years Teaching Experience in Higher Education: Mean (*SD*) | 11.00 (9.5) |
| Total Years Teaching Experience at Boise State University: Mean (*SD*) | 8.03 (7.7) |
| The Year You Graduated with Your Highest Degree: Mean, Mode | 2004, 2013 |
| Age: Mean (*SD*), Min, Max | 43.64 (12.3)  20, 74 |
| Approximately What Percentage of Your Normal Workload Involves Teaching: Mean (*SD*) | 62.51 (30.1) |
| Approximately What Percentage of Your Normal Workload Involves Research: Mean, (*SD*) | 33.02 (23.1) |

Your Highest Academic Degree in Your Primary Discipline (Percentage Responding)

|  |  |
| --- | --- |
| Associate’s degree | 0.8 |
| Bachelor’s degree | 15.6 |
| Master’s degree | 34.8 |
| Education specialist degree | 0.6 |
| Doctoral degree | 43.9 |
| Other | 4.3 |

Academic Status, Gender, and Office Space

|  | Percent Responding |
| --- | --- |
| Academic Status  Tenure / Tenure-track  Non-tenure track | 37.5  62.5 |
| Gender  Male  Female  Prefer not to answer | 40.4  53.9  5.7 |
| Office Space on Campus  Yes  No | 77.6  22.4 |
